# Epidural Anesthesia for Cesarean Section in a Patient With Severe Dilated Cardiomyopathy: A Case Report

**DOI:** 10.7759/cureus.102667

**Published:** 2026-01-30

**Authors:** Marium Amir, Fahd Asif Sahi, Muhammad Sohaib Chaudhary, Ali Nawaz, Syed Sajjad Raza Kazmi

**Affiliations:** 1 Anesthesiology and Critical Care, Shaikh Zayed Hospital, Lahore, PAK; 2 Anesthesiology and Critical Care, Tipperary University Hospital, Clonmel, IRL

**Keywords:** cesarean section (cs), dilated cardiomyopathy (dcm), epidural anesthesia, hemodynamic management, high-risk pregnancy, severe mitral regurgitation

## Abstract

Dilated cardiomyopathy (DCM) in pregnancy presents a significant anesthetic challenge due to compromised myocardial function and limited cardiac reserve. Selection of the anesthetic technique is critical to minimize hemodynamic fluctuations. A 35-year-old gravida 5 para 2+2 at 31 weeks’ gestation with severe intrauterine growth restriction (IUGR) and known dilated cardiomyopathy (ejection fraction 20-25%) underwent elective cesarean section and tubal ligation under epidural anesthesia. Careful titration of local anesthetic, invasive blood pressure monitoring, and multidisciplinary planning ensured hemodynamic stability throughout the procedure.

Epidural anesthesia allows incremental dosing and gradual sympathetic blockade, maintaining preload and afterload within tolerable limits in patients with poor ventricular function. This approach is associated with fewer sudden cardiovascular changes compared to spinal or general anesthesia. Epidural anesthesia can be safely and effectively used for elective cesarean section in non-laboring patients with severe DCM, provided meticulous monitoring and collaborative perioperative management are ensured.

## Introduction

Dilated cardiomyopathy (DCM) is a serious myocardial disorder characterized by left ventricular or biventricular enlargement along with impaired contractility. This results in significant left ventricular systolic dysfunction, characterized by a reduced ejection fraction and progressing to chronic congestive heart failure [1,2]. It has an estimated prevalence of approximately 1 in 2,500 individuals, and nearly half of these cases are classified as idiopathic. Given the high risk of maternal cardiac events in severe cardiomyopathy, management necessitates a tailored, multidisciplinary, and risk-stratified approach to pregnancy counseling. Women with high-risk cardiac lesions who conceive despite recommendations face a >40% risk of serious maternal cardiac complications [3,4]. During pregnancy, maternal cardiac output increases by 30-50%, plasma volume expands, and systemic vascular resistance falls. These hemodynamic demands can exacerbate pre-existing myocardial dysfunction and precipitate heart failure symptoms, particularly during the second trimester and immediate postpartum period [5].

Women with severe dilated cardiomyopathy, particularly those with a left ventricular ejection fraction <40% or New York Heart Association (NYHA) functional class III-IV symptoms, are at high risk of adverse maternal cardiovascular events and mortality during pregnancy, requiring close, specialized, multidisciplinary management. Additional predictors of adverse outcomes include ventricular arrhythmias, rapid atrial fibrillation, severe valvular disease, right ventricular dysfunction, and hypotension [3].

Anesthesiologists are integral in the care of patients with cardiomyopathy, carefully weighing the risks of general anesthesia, which can cause myocardial depression, tachyarrhythmias, and abrupt hemodynamic fluctuations during induction and emergence, against the benefits of regional techniques [6,7]. Early treatment of hypotension (e.g., mean arterial pressure maintained ≥ 65 mmHg) with vasopressor titration and judicious fluid administration is central to preventing decompensation. Graded epidural anesthesia permits incremental sympathetic blockade, attenuates nociceptive-mediated catecholamine surges, and facilitates more stable hemodynamic profiles, thereby reducing the risk of cardiovascular collapse [8]. To optimize maternal and fetal outcomes, clinicians must prioritize precise anesthetic titration, strategic fluid management, and immediate access to vasoactive support [7].

We report a case of an elective cesarean section and tubal ligation in a patient with severe DCM (ejection fraction 20-25%) using a graded epidural anesthesia.

## Case presentation

A 35-year-old female, gravida 5 para 2+2, at 31 weeks’ gestation, was admitted for feto-maternal monitoring due to severe intrauterine growth restriction (IUGR). She was placed on the elective list for cesarean section and tubal ligation. The patient had one living child, two previous miscarriages, and one intrauterine death (IUD). She was a known case of dilated cardiomyopathy for 10 years and had chronic hypertension for five years.

She was under regular cardiology follow-up and was on labetalol 25 mg three times daily, nifedipine 20 mg twice daily, furosemide 20 mg twice daily, and low-molecular-weight heparin (LMWH) 60 mg subcutaneously twice weekly to prevent venous thromboembolism and reduce the risk of left ventricular thrombus formation caused by low blood flow. Her symptoms were well controlled, and she was categorized as NYHA Class II [9].

**Figure 1:**
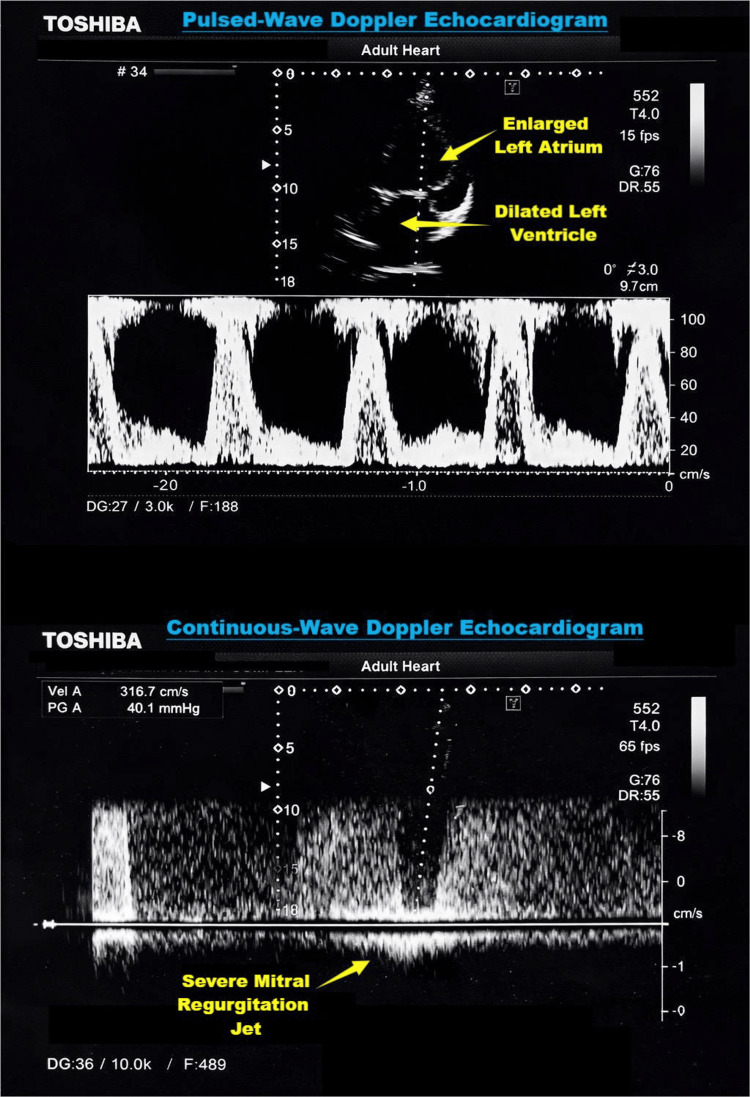
Transthoracic echocardiographic Doppler findings showing severe mitral regurgitation with left atrial enlargement and left ventricular dilatation The upper panel demonstrates pulsed-wave Doppler imaging, while the lower panel shows continuous-wave Doppler demonstrating a severe mitral regurgitant jet.

**Figure 2:**
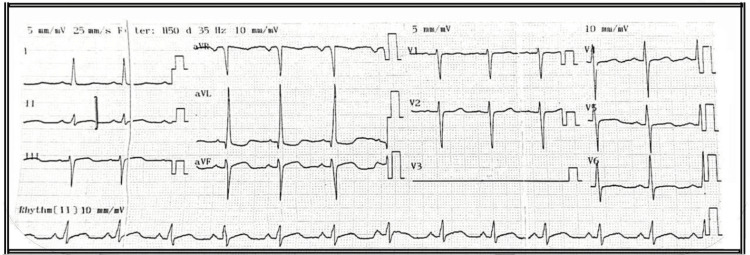
Preoperative electrocardiogram showing sinus rhythm with low-voltage QRS complexes, poor R-wave progression in chest leads (V1-V6), left axis deviation, and nonspecific ST-T changes suggestive of diffuse myocardial disease

Echocardiography revealed severe dilated cardiomyopathy with marked left ventricular dilatation, a severely reduced ejection fraction (20-25%), grade III diastolic dysfunction, severe global hypokinesia, severe mitral regurgitation, tricuspid regurgitation, aortic regurgitation, bi-atrial enlargement, and overall impaired left ventricular systolic function (Figure 1). Chest X‑ray was unremarkable. Laboratory investigations showed a hemoglobin of 13.0 g/dL, and serum electrolytes, renal, and liver function tests were within normal limits. The international normalized ratio (INR) was 0.9. Preoperative ECG showed sinus rhythm with low-voltage QRS complexes, poor R-wave progression in chest leads (V1-V6), left axis deviation, and nonspecific ST-T changes suggestive of diffuse myocardial disease (Figure 2).

On physical evaluation, her heart rate was 80 beats per minute, blood pressure was 153/115 mmHg, and oxygen saturation was 97% at room air. On auscultation, an audible systolic ejection murmur was heard. No bilateral pedal edema was observed. A multidisciplinary team involving obstetricians, anesthesiologists, and cardiologists discussed and optimized the patient preoperatively. LMWH dosage was verified for a safe neuraxial block as the last dose was given 12 hours prior to the procedure.

The anesthetic plan aimed to achieve a gradual sympathetic block to prevent sudden hypotension, maintain stable preload, and avoid myocardial depression. A backup plan of general anesthesia was in place in case of epidural failure. After obtaining high-risk informed written consent, the patient was transferred to the operating theatre. Two 16-G intravenous lines were secured, and standard ASA (American Society of Anesthesiologists) monitoring (pulse oximetry, NIBP, ECG) was established. Baseline vitals were: heart rate 92 bpm, blood pressure 142/102 mmHg, and oxygen saturation 98% on room air. The patient was co-loaded with a 300 mL bolus of normal saline (0.9%), and the left radial artery was cannulated for continuous invasive blood pressure monitoring after local infiltration of 2 mL of 2% lidocaine. Vasopressors (ephedrine, phenylephrine, and norepinephrine) and inotropes (dobutamine) were prepared for immediate use, a defibrillator was kept on standby with stat pads applied prophylactically, and intensive care unit (ICU) backup was ensured.

Under strict aseptic technique, with the patient in a sitting position, 4 mL of 2% lidocaine was used for local infiltration at the L3-4 intervertebral space. The epidural space was identified at 5 cm using an 18-G Tuohy needle with loss of resistance to air. A test dose of 3 mL of 2% lidocaine with adrenaline was administered to rule out intravascular and intrathecal placement. The catheter was secured at 10 cm. The procedure was uneventful, and the patient was positioned supine with lateral uterine displacement using a wedge under the right hip to minimize aortocaval compression. A dose of 10 mL of 2% lidocaine with adrenaline (1:200,000), followed by 5 mL of 0.5% bupivacaine, was given in the epidural space, incrementally as a bolus of 2-3 ml of local anesthetic each time to observe the graded response. Sensory and motor blocks were evaluated, and after establishing a successful sensory blockade at the T4 level, surgery commenced.

Oxygen was administered at 5 L/min via a Hudson facemask, and a baby girl weighing 0.8 kg was delivered in an extended breech presentation. She was admitted to the neonatal intensive care unit (NICU) for further management. The amniotic fluid was clear, and the placenta and membranes were delivered intact. After umbilical cord clamping, a bolus of 2+2 IU of oxytocin was administered slowly due to its potential to cause tachycardia and hypotension, followed by a continuous infusion of 40 IU/1,000 mL normal saline at a rate of 75 ml/h (equivalent to 3 IU/h). The APGAR scores were 6 and 8 at 1 and 5 minutes, respectively. Vigilant monitoring was performed to detect early signs of autotransfusion-related flash pulmonary edema due to rapid uterine contraction and return of blood volume, and a continuous positive airway pressure (CPAP) machine was kept ready for immediate respiratory support if necessary.

Intraoperatively, hemodynamic stability was maintained compared to baseline values, and no vasopressors were required. Urine output remained adequate (150 mL). A repeat dose of 5 mL of 0.5% bupivacaine was given toward the end of the procedure. Bilateral tubal ligation was performed. The total intraoperative fluid given was 900 mL of normal saline, the estimated blood loss was 300 mL, and the total surgical duration was 50 minutes.

Postoperatively, the patient was transferred to the ICU for 24-hour monitoring with continuous electrocardiogram (ECG), pulse oximetry, and invasive blood pressure measurement. An infusion of 0.1% bupivacaine was initiated at 7 mL/hour for postoperative pain management, and the dosage was adjusted based on sensory levels. Judicious fluid balance was maintained to prevent volume overload, ensuring adequate input/output control. Cardiology consultation was obtained, and furosemide was reduced to 20 mg once daily, while other medications were continued. The patient remained hemodynamically stable. The epidural catheter was removed on the first postoperative day with the blue tip intact, and the patient was transitioned to systemic analgesics. She was transferred to the ward after 24 hours with no perioperative complications and discharged home in stable condition two days later.

## Discussion

The anesthetic management of obstetric patients with dilated cardiomyopathy (DCM) poses a significant challenge, as even small hemodynamic disturbances can precipitate cardiac decompensation. The key perioperative concerns for management are to maintain myocardial contractility, avoid tachycardia and arrhythmias, maintain preload, and prevent abrupt reductions in afterload [10-11]. 

Among the available anesthetic techniques, epidural anesthesia offers the advantage of a gradual onset and an easily titrated, efficient sympathetic blockade, making it especially suitable for patients with limited cardiac reserve [12-14]. Incremental or titrated dosing can minimize sudden alterations in systemic vascular resistance and venous return, thereby maintaining adequate systemic and coronary perfusion. This contrasts with spinal anesthesia, which produces rapid onset sympathectomy and marked hypotension, which can be catastrophic in patients with severe DCM [12]. General anesthesia remains an alternative in patients with contraindications to regional anesthesia or when patient cooperation is limited. However, it comes with risks of myocardial depression, increased sympathetic stimulation during intubation, and potential arrhythmias. Volatile anesthetics and induction agents should be chosen carefully to avoid negative inotropic effects, while ensuring adequate depth to suppress stress responses [11,15]. 

Continuous invasive monitoring is indispensable. In this case, arterial line monitoring enabled early detection and management of pressure fluctuations. While central venous or pulmonary artery catheterization may provide additional data on volume status and cardiac output, they are not routinely required unless instability is anticipated [13]. Excessive preload can worsen pulmonary congestion and could present as flash pulmonary oedema, whereas hypovolemia can reduce cardiac output. Therefore, judicious fluid administration is essential. Avoidance of fluid overload is particularly important in patients with elevated filling pressures and impaired left ventricular compliance [11,14].

Vasopressors should be used cautiously. Agents such as phenylephrine can increase afterload and reduce cardiac output, while ephedrine may cause tachycardia and exacerbate myocardial oxygen demand. Low-dose norepinephrine infusion is sometimes preferred for maintaining perfusion without an excessive chronotropic effect [14]. Oxytocin, though routinely administered after delivery, can cause vasodilation, hypotension, and tachycardia if given rapidly and thus should be used in low doses and cautiously. Current evidence recommends a slow intravenous bolus (≤5 IU) followed by a low-dose infusion to minimize hemodynamic instability [13,15]. Ergometrine and prostaglandin F₂α are contraindicated due to their hypertensive and cardio-toxic potential [14]. Postoperative management should focus on pain control, fluid balance, and continued cardiac monitoring. Epidural analgesia with dilute local anesthetic provides stable hemodynamic and excellent pain relief without respiratory depression. ICU observation for at least 24 hours allows early detection of arrhythmias, pulmonary oedema, or heart failure exacerbation [11,13]. Successful outcomes in such high-risk obstetric patients depend on collaboration between anesthesiology, cardiology, obstetrics, and intensive care teams. Preoperative optimization, intraoperative vigilance, and postoperative surveillance are critical in preventing decompensation [16,17]. 

Our patient tolerated the epidural technique well, with stable intraoperative hemodynamic parameters and no need for vasopressors. The avoidance of large fluid boluses, slow oxytocin administration, and postoperative ICU observation contributed to a favorable outcome. Elective planning and hemodynamic vigilance were critical to our success, particularly when compared to the high-risk emergency scenario described by Thakur et al. [15].

## Conclusions

Anesthetic management of patients with severe dilated cardiomyopathy requires vigilant hemodynamic control strategies and multidisciplinary team coordination. This case demonstrates that graded epidural anesthesia can provide stable cardiovascular conditions and satisfactory surgical anesthesia for caesarean delivery in high-risk, non-laboring patients with poor ventricular function.

Careful fluid management, invasive monitoring, and slow oxytocin administration are essential for preventing cardiac decompensation. With meticulous planning and multidisciplinary collaboration between anesthesiology, cardiology, and obstetrics, epidural anesthesia remains a safe and effective choice for caesarean section in women with advanced DCM.
